# Software for the Diagnosis of Sarcopenia in Community-Dwelling Older Adults: Design and Validation Study

**DOI:** 10.2196/13657

**Published:** 2020-04-13

**Authors:** Lydia Lera, Bárbara Angel, Carlos Márquez, Rodrigo Saguez, Cecilia Albala

**Affiliations:** 1 Public Health Nutrition Unit Institute of Nutrition and Food Technology University of Chile Santiago de Chile Chile

**Keywords:** sarcopenia, software, elderly, muscle, mHealth

## Abstract

**Background:**

The usual diagnosis of sarcopenia requires a dual-energy x-ray absorptiometry (DXA) exam, which has low accessibility in primary care for Latin American countries.

**Objective:**

The aim of this study is to design and validate software for mobile devices (Android, IOS) and computers, based on an adapted version of the diagnostic algorithm of sarcopenia proposed by the European Working Group on Sarcopenia in Older People (EWGSOP).

**Methods:**

Follow-up exams were conducted on 430 community-dwelling Chileans 60 years and older (mean 68.2 years, SD 4.9) participating in the IsaMayor and Alexandros cohorts designed to study sarcopenia and disability associated with obesity, respectively. All the participants from the cohorts were randomly selected from the registries of primary health care centers and, for this study, must have a DXA scan at baseline. The software (HTSMayor) was designed according to an adapted version of the algorithm proposed by the EWGSOP and was divided into four phases: longitudinal validation of diagnostic algorithm of sarcopenia, alpha version, beta version, and release version. The software estimates appendicular skeletal muscle mass (ASM) using an anthropometric equation or DXA measurements with Chilean cut-off points. The predictive validation of the algorithm was estimated, comparing functional limitations (at least one activity of daily living, two instrumental activities of daily living, or three mobility limitations), falls, and osteoporosis at follow-ups in patients with and without sarcopenia at baseline, using adjusted logistic models.

**Results:**

After a median follow-up of 4.8 years (2078.4 person-years), 37 (9.9%) new cases of sarcopenia, out of the 374 patients without sarcopenia at baseline, were identified (incidence density rate=1.78 per 100 person-years). ASM estimated with the anthropometric equation showed both a high sensitivity and specificity as compared with those estimated by DXA measurements, yielding a concordance of 0.96. The diagnostic algorithm of sarcopenia considered in the software with the equation showed both a high sensitivity (82.1%) and specificity (94.9%) when compared with DXA (reference standard). Adults without sarcopenia (at baseline) showed better physical performance (after approximately 5 years) than adults with sarcopenia. Loss of functionality was greater in adults with sarcopenia (OR 5.0, 95% CI 2.2-11.4) than in adults without sarcopenia. In addition, the risks of falls (OR 2.2, 95% CI 1.1-4.3) and osteoporosis (OR 2.8, 95% CI 1.2-6.6) were higher in older persons with sarcopenia than those without sarcopenia. The measurements and results were completed for the beta and release tests with a mean time of 10 minutes and 11 minutes, respectively.

**Conclusions:**

We developed and validated a software for the diagnosis of sarcopenia in older Chilean adults that can be used on a mobile device or a computer with good sensitivity and specificity, thus allowing for the development of programs for the prevention, delay, or reversal of this disease. To our knowledge, HTSMayor is the first software to diagnose sarcopenia.

**International Registered Report Identifier (IRRID):**

RR2-10.2196/13657

## Introduction

The accelerated process of demographic and epidemiological transition occurring globally in recent decades accompanies a progressive aging of the population and an increase in the frequency of chronic diseases [1], which subsequently increases the burden of disease, evidenced by an increase in disability-adjusted life years lost [2].

Sarcopenia, a disease characterized by the progressive loss of muscle mass and skeletal muscle strength, is one of the pathologies that most affects older people and has serious consequences on health, such as increases in falls, fractures, disabilities, institutionalization, poor quality of life, and mortality [3-11]. Since October 1, 2016, the International Classification of Disease, tenth revision, clinical modification defines sarcopenia as a disease (M62.84) [12,13].

In 2010, the European Working Group on Sarcopenia in Older People (EWGSOP) [3] developed a consensus diagnostic criterion by means of a diagnostic algorithm, which was revised in 2018. It is based on measurements of gait speed, handgrip strength, and appendicular skeletal muscle mass (ASM) measured by dual-energy x-ray absorptiometry (DXA), as well as chair-stands, which was added in 2018 [14]. Sarcopenia is highly prevalent [9] with ranges between 4% and 32.8% [15], and reaching 50% in people 80 years and older [3]. The increase in life expectancy and the rapid increase in the 80 years and older population predicts an increase in the prevalence and adverse consequences of sarcopenia [16]; therefore, it is important to include its diagnosis in routine preventive medical exams.

Even though the identification of sarcopenia is a key issue in preventing its negative effects on health and there is agreement on the need for widespread screening and treatment for sarcopenia in older people [14,17], the usual diagnosis of sarcopenia requires a DXA exam, which is scarcely accessible and expensive not only in Latin America [17] but also in developed countries [18-24]. Furthermore, access to the test has been associated with education and income level [23,24].

The low accessibility and high cost of current diagnostic tools evidences the need for screening tools with diagnostic methods that are easily accessible and inexpensive at the primary health care level. Considering the high prevalence of sarcopenia in Chile, 19.1% in individuals 60 years and older and 38.5% in individuals 80 years and older [25], and the importance of its early diagnosis for preventing adverse consequences, we developed and validated an anthropometric prediction equation for muscle mass estimation for the screening of sarcopenia (as an alternative to DXA measurements). In addition, we also validated the EWGSOP algorithm for the identification of sarcopenia in older Chileans [26,27].

The aim of this study was to design and validate a computer-based software, which can also be used on mobile devices, that allows the use of either a DXA exam or the anthropometric prediction equation for the diagnosis of sarcopenia in older Chileans at primary health care centers [25], according to the validated algorithm of the EWGSOP [3].

## Methods

### Design and Participants

Follow-ups were conducted with 430 community-dwelling people 60 years and older (mean years of age 68.2, SD 4.9; 299 females, 69.7%) living in Santiago de Chile, with baseline measurement of body composition by DXA scan from the IsaMayor and Alexandros cohorts designed to study sarcopenia and functionality, respectively [27,28]. Baseline data were collected between 2012 and 2013, and the second measurement was done in 2017, with a median follow-up time of 4.8 years (range 3-5 years).

The study and the informed consent form were approved by the Ethics Committee of the Institute of Nutrition and Food Technology at the University of Chile. Before any procedures were performed, all subjects signed the consent form.

### Data Collection

After signing an informed consent, all subjects underwent face-to-face interviews, which included questions on self-reported chronic diseases and self-reported functional limitations. Functional status was determined by a self-report of the ability to perform six activities of daily living (ADL), six instrumental activities of daily living (IADL), and seven mobility limitations. Multimorbidity was defined as having two or more chronic diseases [29].

Anthropometric measurements including weight (kg), height (cm), knee height (cm), calf circumference (cm), hip circumference (cm), and handgrip strength (kg) were performed according to the methods described previously [30]. Handgrip strength was measured by means of a handgrip dynamometry (JAMAR dynamometer), registering the best of two measurements with the dominant hand, according to a previously described technique [31].

A DXA scan to assess body composition was performed for the whole sample at the beginning and at the end of the study. The skeletal muscle mass index (SMI) was calculated as the ratio of ASM to the height squared (kg/m^2^). ASM was estimated by the anthropometric prediction equation [27] and by DXA scan (reference standard) [32].

Low muscle mass was defined with the cut-off points obtained for the Chilean population using DXA measurements or the anthropometric prediction equation with Chilean cut-off points (DXA: ≤7.19 kg/m^2^ for men and ≤5.77 kg/m^2^ for women; equation: ≤7.45 kg/m^2^ for men and ≤5.88 kg/m^2^ for women). Low muscle strength was defined with cut-off points previously determined in a large sample of the older adult Chilean population (≤25th percentile: 27 kg for men; 15 kg for women) [31,33]. Low physical performance was defined with the 3-meter gait speed test using the same cut-off points defined by the EWGSOP (0.8 m/sec) or for the five chair-stand test (>10 sec) [34] when the gait speed test could not be performed. The prediction equation for men and women is shown in Textbox 1.

**Prediction equation for men and women.**

**Men**

ASM (kg) = 0.107 * weight + 0.251 * knee height + 0.047 * handgrip strength – 0.02 * age – 0.034 * hip circumference – 4.228

**Women**

ASM (kg) = 0.107 * weight + 0.251 * knee height + 0.047 * handgrip strength – 0.02 * age – 0.034 * hip circumference – 7.646

**Goodness of fit of the model**

*R*^2^=0.89; standard error of estimate=1.346

### Design of the Software HTSMayor

The software uses an adapted version of the diagnostic algorithm of sarcopenia proposed by the EWGSOP [25]. ASM can be estimated by DXA scan (when available) or with the anthropometric prediction equation previously described. When the gait speed test could not be performed, the algorithm used the five chair-stand test. The cut-off points for the SMI and handgrip strength were specific to the Chilean population.

**Figure 1:**
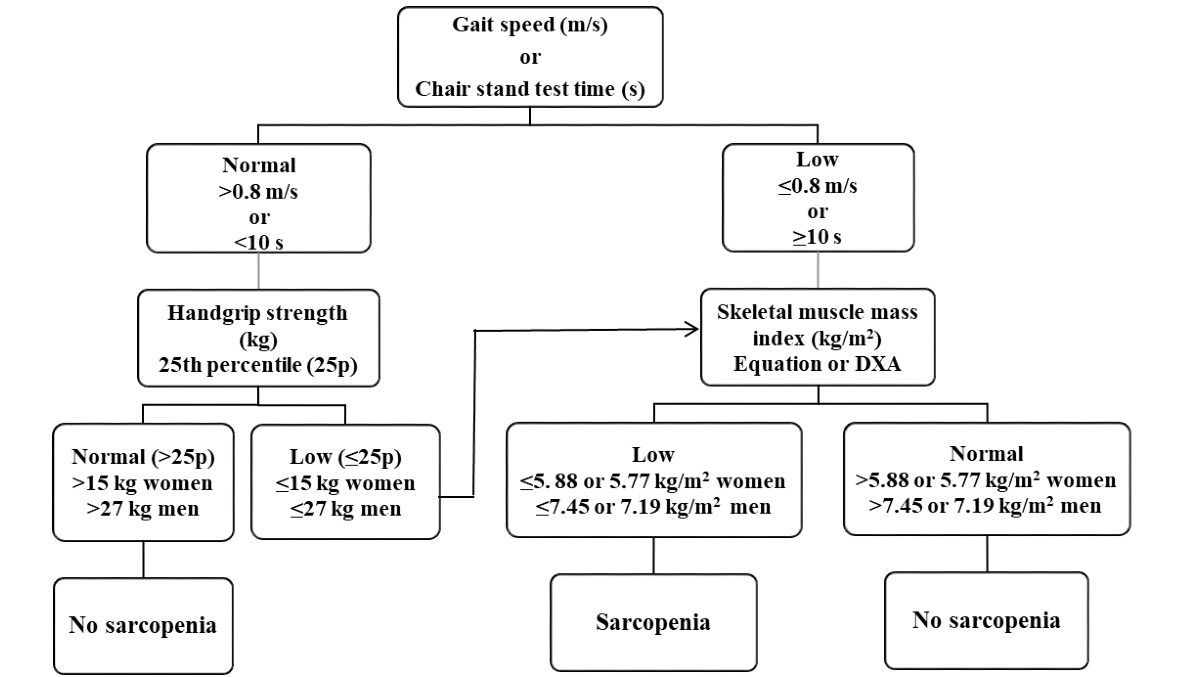
HTSMayor diagnostic algorithm for sarcopenia.

The software starts by asking for age and sex, then if the patient has taken a DXA test. If the answer is negative, it asks for the physical performance test used (the 3-meter gait speed test or five chair-stand test). Then, it asks for the anthropometric measurements (weight, height, knee height, calf circumference, and hip circumference) and handgrip strength to estimate the ASM in kg using an anthropometric prediction equation. Otherwise, the ASM is calculated through DXA measurements [32,35] of the muscle mass of the right and left arms and legs in kg, following the diagnostic algorithm in Figure 1.

The final outcomes of the software were presarcopenia (low muscle mass), sarcopenia (low muscle mass and low muscle strength or low physical performance) and severe sarcopenia (low muscle mass, low muscle strength, and low physical performance), according to the suggested classification of the EWGSOP [3].

### Main Outcome Measures

Functionality was defined according to the criteria that Albala et al [36] (2004) proposed for the older Chilean adult population, namely, limitation in at least one ADL, two IADL, or three mobility limitation questions, as well as self-reported falls in the last year.

Sarcopenia was defined according to an adapted version of the algorithm of the EWGSOP [25]. World Health Organization (WHO) standards for bone mineral density were used for the identification of osteoporosis.

### Development and Validation of the Software

**Figure 2:**
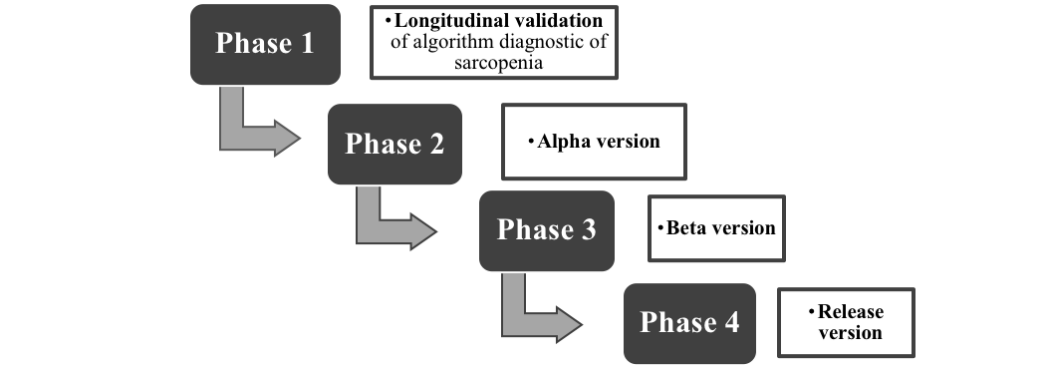
Phases of the software.

The development and validation of the software can be divided into four phases (Figure 2).

#### Phase 1: Longitudinal Validation

In this phase, the calculation of the diagnostic accuracy of the diagnostic algorithm of sarcopenia, with the ASM estimated by the anthropometric equation using DXA as a reference standard, and the predictive validation of the adapted version of the diagnostic algorithm of sarcopenia were performed. The predictive validation of the diagnostic algorithm was performed for the outcomes of functional limitations, ADL, IADL, mobility limitations, falls, and osteoporosis at follow-up in patients with sarcopenia and without sarcopenia at baseline.

#### Phase 2: Alpha Version

Considering the validated diagnostic algorithm, a programmer completed an initial design of a prototype of the software for the web and mobile apps. The software was developed to run on all platforms, including Android, IOS, and the Web, and used Java, Swift, and PHP, respectively, as the programming languages.

Based on the diagnostic algorithm, the software (HTSMayor) Multimedia Appendix 1 estimated if a person had sarcopenia or not, and, if sarcopenic, the stage of sarcopenia—presarcopenia, sarcopenia, and severe sarcopenia—was shown. The icons for the app and the web version as well as the stages of sarcopenia calculated by the software are shown in Multimedia Appendix 2. The software generated a Microsoft Excel file with the variables measured and calculated.

#### Phase 3: Beta Version

A version of the software using the initial design and the same platform was developed. In this phase, the beta test of the software was completed and applied to 128 older adults (29 men and 99 women) living in the communities registered at seven centers of primary health care in three regions of Chile (Metropolitan Region, V Region, and XV Region) as a pilot study. Then, some changes were made to the beta version to transform it in the release version. Anthropometric measurements, physical performance tests, and the use of HTSMayor were carried out by paramedical specialized personnel trained for this study.

#### Phase 4: Release Version

In this phase, a validation of HTSMayor was performed by the medical team in 48 public health care centers in five regions of Chile (Metropolitan region, V region, VIII region, IX region, and XV region) and in the National Institute of Geriatrics of Santiago de Chile in a sample of 4242 community-dwelling people 60 years and older (979 men and 3263 women) served by public health care centers.

Finally, this phase led to the creation of a final version 2.0 of HTSMayor, which will be delivered to the Ministry of Health of Chile (MINSAL); this entity will be responsible for promoting the use of the software in primary health care centers.

### Statistical Analysis

Continuous variables were expressed as mean (SD) or the medians and interquartile ranges with a 95% CI. Categorical variables were expressed as percentages and 95% CI. The difference between sexes was calculated by a two-sample mean comparison test or Pearson’s chi-square test, depending on the type of variable. Differences between DXA measurements and equation estimations were estimated by two-sample tests for paired data. The prevalence of sarcopenia was compared by Cohen kappa coefficient and McNemar’s test. Differences in physical functionality at follow-up among patients with and without sarcopenia, diagnosed at baseline with DXA and the anthropometric equation, were compared by two-sample tests for unpaired and paired data. Relative risk was also calculated. Sensitivity, specificity, positive and negative likelihood ratios, and positive and negative predictive values of sarcopenia diagnosed by DXA and the equation were calculated. In addition, the incidence density rate was calculated. Lin's concordance correlation coefficient was calculated to measure the agreement between the diagnostic algorithm of sarcopenia with the ASM estimated by the anthropometric equation and by DXA as a reference standard. Logistic regression models were performed to predict functional limitations, falls, and osteoporosis with sarcopenia diagnosed at baseline with HTSMayor (prediction validation), adjusted by age, sex, nutritional state, and lean mass/fat mass ratio. The Hosmer-Lemeshow test was used to assess the goodness of fit for the estimated models.

## Results

### Phase 1

Table 1 shows the sociodemographic and health characteristics of the study sample at baseline by sex. The mean age of the sample was 68.2 years of age (SD 4.9; range 60-88). The years of education, ADL and IADL limitations, fractures, and BMI were similar in both sexes. The proportion of women living alone was higher than in men. Falls and multimorbidity were higher in women than in men. Gait speed, anthropometric variables, and body composition were higher in men than in women, with a lean mass/fat mass ratio almost double in the former.

**Table 1:** Participants characteristics by sex.

Characteristics		Men (N=131)		Women (N=299)		Total (N=430)		*P* value^a^	
Age (years), mean (SD)		68.7 (5.3)		67.9 (4.7)		68.2 (4.9)		.14	
Education (years; n=291, 94 men and 197 women), mean (SD)		9.0 (4.3)		9.5 (4.5)		9.3 (4.5)		.34	
Living alone, n (%)		6 (4.6)		33 (11.0)		39 (9.1)		.03	
Smoking, n (%)		15 (11.4)		18 (6.1)		33 (7.7)		.05	
Functional limitation in one ADL^b^, n (%)		21 (16.0)		56 (18.7)		77 (17.9)		.49	
Functional limitation in two IADL^c^, n (%)		5 (3.8)		8 (2.7)		13 (3.0)		.52	
Functional limitation in three mobility activities, n (%)		5 (3.8)		30 (10.0)		35 (8.1)		.03	
Multimorbidity, n (%)		62 (47.3)		204 (68.2)		266 (61.9)		<.001	
Falls, n (%)		24 (18.3)		91 (30.4)		115 (26.7)		.01	
Fractures, n (%)		16 (12.2)		58 (19.4)		74 (17.2)		.07	
BMI (kg/m^2^), mean (SD)		29.0 (4.8)		29.7 (5.6)		29.5 (5.4)		.20	
**Nutritional state, n (%)**									.48
	Underweight (BMI<20)		2 (1.5)		3 (1.0)		5 (1.2)		
	Normal (BMI 20-24.9)		24 (18.3)		62 (20.7)		86 (20.0)		
	Overweight (BMI 25-29.9)		57 (43.5)		108 (36.1)		165 (38.4)		
	Obese (BMI≥30)		48 (36.6)		126 (42.1)		174 (40.5)		
Calf circumference (cm), mean (SD)		37.0 (3.2)		35.5 (3.4)		36.0 (3.4)		<.001	
Knee height (cm), mean (SD)		51.8 (2.7)		47.4 (2.2)		48.8 (3.1)		<.001	
Waist circumference (cm), mean (SD)		101.4 (11.8)		94.7 (12.9)		96.7 (12.9)		<.001	
Hip circumference (cm), mean (SD)		102.1 (9.4)		105.8 (11.4)		104.7 (10.9)		.001	
Handgrip strength (kg), mean (SD)		34.8 (8.5)		20.2 (5.6)		24.6 (9.5)		<.001	
Lean mass (kg), mean (SD)		51.1 (6.6)		36.6 (5.2)		41.0 (8.8)		<.001	
Lean mass/fat mass, mean (SD)		2.4 (1.4)		1.3 (0.4)		1.7 (1.0)		<.001	
Gait speed (m/sec), mean (SD)		0.9 (0.2)		0.8 (0.2)		0.8 (0.2)		<.001	

^a^Based on *t* test, except categorical variables, which were based on Pearson chi-square test.

^b^ADL: activities of daily living.

^c^IADL: instrumental activities of daily living.

Table 2 shows the sensitivity, specificity, positive predictive value, negative predictive value, positive likelihood ratio, and negative likelihood ratio for the diagnostic algorithm of sarcopenia with the values estimated by the prediction equation using DXA as the reference standard. The mean ASM measured with DXA or estimated with the prediction equation was similar for men, women, and the whole sample. The frequency of sarcopenia estimated with DXA or with the equation were similar (McNemar’s test: *P*=.27; agreement=93.3% and kappa statistic=0.74; *P*<.001). The prediction equation used by the software for the diagnosis of sarcopenia had a high sensitivity (82.1%; higher in men than in women, 95% vs 75%, respectively) and better specificity (94.9%), which was similar in both men and women when compared with DXA, yielding a concordance of 0.955. Positive and negative predictive values were higher in men than in women. In men, the positive and negative likelihood ratios indicated that it was almost 35 times more likely to obtain a positive diagnosis in sick patients than in healthy ones and that the probability of obtaining a negative diagnosis is 19.5 times more likely in healthy patients than in sick patients. In women, it was 12 times more likely to obtain a positive diagnosis in sick patients than in healthy ones, and the probability of obtaining a negative diagnosis in healthy patients was almost four times (3.8 times) more likely than in sick patients.

**Table 2:** Diagnostic accuracy of the diagnostic algorithm for sarcopenia with ASM estimated by the anthropometric equation and using DXA as the reference standard.

Variables		Men (N=131)		Women (N=299)		Total (N=430)
**ASM^a^ (kg), Lin’s concordance^b^, mean (SD, 95% CI)**						
	DXA^c^		21.4 (3.3, 20.8-21.9)		14.8 (2.4, 14.5-15.1)	16.8 (4.1, 16.4-17.2)
	Equation		21.5 (2.6, 21.0-21.9)		14.7 (2.2, 14.5-15.0)	16.8 (3.9, 16.4-17.1)
**SMI^d^ (kg/m^2^), Lin’s concordance, mean (SD, 95% CI)**						
	DXA		7.92 (1.1, 7.7-8.1)		6.50 (0.9, 6.4-6.6)	6.9 (1.2, 6.8-7.0)
	Equation		7.97 (0.8, 7.8-8.1)		6.47 (0.8, 6.4-6.6)	6.9 (1.1, 6.8-7.0)
**Sarcopenia, % (95% CI)**						
	DXA		17.6 (11.5-25.2)		13.7 (10.0-18.1)	14.9 (11.7-18.6)
	Equation		18.3 (12.1-26.0)		15.4 (11.5-20.0)	16.3 (12.9-20.1)
**Summary statistics for diagnostic tests (equation) compared to true disease status (DXA)**						
	Sensitivity, % (95% CI)		95 (75.1-99.9)		75 (57.8-87.9)	82.1 (69.6-91.1)
	Specificity, % (95% CI)		97.3 (92.3-99.4)		93.9 (90.3-96.5)	94.9 (92.2-96.9)
	PPV^e^, % (95% CI)		86.4 (65.1-97.1)		62.8 (46.7-77)	70.8 (58.2-81.4)
	NPV^f^, % (95% CI)		99.1 (95-100)		96.5 (93.4-98.4)	97.3 (95.0-98.7)
	Positive likelihood ratio, % (95% CI)		35.1 (11.5-98)		12.3 (7.4-20.5)	16.2 (10.3-25.5)
	Negative likelihood ratio, % (95% CI)		0.05 (0.01-0.35)		0.27 (0.15-0.47)	0.19 (0.11-0.33)

^a^ASM: appendicular skeletal muscle mass.

^b^Lin’s concordance: Lin’s concordance correlation coefficient was 0.96.

^c^DXA: Dual-energy x-ray absorptiometry.

^d^SMI: appendicular skeletal muscle mass index.

^e^PPV: positive predictive value.

^f^NPV: negative predictive value.

After a median follow-up of 4.8 years (2078.4 person-years), there were 37 (9.9%) new cases of sarcopenia out of the 374 people without sarcopenia at baseline, who were identified by means of a DXA scan (incidence density rate=1.8 per 100 person-years).

Table 3 presents the longitudinal predictive validation of the diagnostic algorithm. Six logistic regression models for the association of ADL, IADL, mobility limitations, functional limitations, falls, and osteoporosis at the end of the follow-up according to the presence of sarcopenia at baseline (diagnosed by HTSMayor) were performed. After adjusting for sex, age, nutritional status, lean mass/fat mass ratio, and morbidity in all models, adults with sarcopenia had a higher risk of presenting adverse conditions than robust adults. The loss of functionality, mobility, and ADL were greater in adults with sarcopenia. In addition, the risk of falls and osteoporosis were higher in older persons with sarcopenia as compared to older persons without sarcopenia. A total of 5 people with BMI<20 were removed from the regressions.

**Table 3:** Logistic regression models with functionality, falls, and osteoporosis adjusted by age, sex, nutritional state, lean mass/fat mass ratio, and sarcopenia diagnosis at baseline.

Baseline variables^a^		Follow-up									
		ADL^b^		IADL^c^		Mobility limitation		Functional limitation		Falls	Osteoporosis
Sarcopenia, OR^d^ (95% CI)		4.4 (1.6-12.1)		3.7 (1.1-12.6)		4.4 (1.9-10.4)		5.0 (2.2-11.4)		2.2 (1.1-4.3)	2.8 (1.2-6.6)
Women, OR (95% CI)		1.4 (0.5-3.8)		0.6 (0.2-2.0)		0.6 (0.3-1.2)		0.7 (0.4-1.5)		1.2 (0.7-2.3)	55.2 (8.0-379.2)
**Age (years), OR (95% CI)**											
	70-79	2.5 (1.2-5)	1.6 (0.7-4.1)		0.9 (0.5 -1.6)		1.3 (0.7-2.1)		0.7 (0.5-1.2)		1.8 (0.9-3.5)
	≥80	5 (1.2-21.8)	1.4 (0.1-14.1)		0.9 (0.2-5.2)		3.3 (0.8-14)		0.7 (0.2-2.4)		1.6 (0.3-9.4)
**Nutritional state (kg/m^2^), OR (95% CI)**											
	Overweight (BMI 25-29.9)	1.5 (0.5-4.8)	1.1 (0.3-4.7)		0.9 (0.4-2.4)		1.7 (0.7-4.1)		1.1 (0.5-2.1)		1.4 (0.5-3.5)
	Obese (BMI≥30)	3.0 (0.8-11.7)	1.3 (0.2-7.2)		2.1 (0.7-6.3)		3.5 (1.3-9.8)		1.5 (0.7-3.3)		1.2 (0.4-3.6)
Lean mass/fat mass ratio, OR, (95% CI)		1.0 (0.5-2.2)		0.8 (0.3-2.2)		0.5 (0.2-1.1)		0.7 (0.3-1.3)		1.0 (0.6-1.7)	3.1 (1.5-6.4)
Multimorbidity (≥2 diseases), OR (95% CI)		1.0 (0.5-2.1)		1.4 (0.5-3.8)		2.2 (1.2-3.9)		1.9 (1.1-3.2)		1.1 (0.7-1.8)	1.1 (0.6-2.2)
Hosmer-Lemeshow test^e^, *P* value		.94		.79		.53		.79		.80	.71

^a^Men, normal nutritional state, and having 0-1 chronic diseases were used as reference categories.

^b^ADL: activities of daily living.

^c^IADL: instrumental activities of daily living.

^d^OR: odds ratio.

^e^Hosmer-Lemeshow test indicated the goodness of fit of the models are satisfactory.

### Phase 3

In the beta test, the total time per patient was approximately 10 minutes, including the time needed to make the measurements and type them into the app to get the diagnosis. The beta test demonstrated the viability of the software. We found that 17.2% (22/128) of adults in this sample had sarcopenia, and 4.7% (6) of them had severe sarcopenia.

Two changes were made to the beta version to transform it in the release version—the inclusion of cut-off points for the chair-stand test and the identification of the patient.

### Phase 4

The mean time required for the software application per patient at the health services was 11 minutes, which was similar to the beta test.

Table 4 shows the sociodemographic and health characteristics of the release study sample at this phase by sex. The average age was higher in men than in women (74.75 years vs 72.58 years, respectively) and ranged from 60 to 92 years, with 76.92% of the sample being women. Body composition variables and anthropometric variables were significantly higher in men than in women (*P*<.001); although there was no difference between average gait speed (3-meter walking speed) in both sexes.

Out of 4242 participants, 18.36% (779) had presarcopenia and 24.21% (1027) had sarcopenia (755, 17.80% with sarcopenia and 272, 6.41% with severe sarcopenia).

The release test also demonstrated the viability of the software.

**Table 4:** Release version: participant characteristics by sex.

Characteristics		Men, (N=979) (23.08%)	Women, (N=3263) (76.92%)	Total (N=4242)	*P* value^a^
Age (years), mean (SD, 95% CI)		74.75 (6.13, 74.36-75.13)	72.58 (6.61, 72.35-72.81)	73.08 (6.56, 72.88-73.28)	<.001
BMI (kg/m^2^), mean (SD, 95% CI)		28.24 (4.39, 27.96-28.51)	29.46 (5.73, 29.26-29.66)	29.18 (5.48, 29.01-29.34)	<.001
**Nutritional state, n (** **%)**					<.001
	Underweight (BMI<20 kg/m^2^)	5 (0.57)	14 (0.47)	19 (0.50)	
	Normal (BMI 20-24.9 kg/m^2^)	185 (21.05)	540 (18.31)	725 (18.94)	
	Overweight (BMI: 25-29.9 kg/m^2^)	418 (47.55)	1194 (40.49)	1612 (42.11)	
	Obese (BMI≥30 kg/m^2^)	271 (30.83)	1201 (40.73)	1472 (38.45)	
Calf circumference (cm), mean (SD, 95% CI)		36.90 (3.64, 35.91-36.16)	36.04 (3.64, 35.91-36.16)	36.24 (3.64, 36.13-36.35)	<.001
Knee height (cm), mean (SD, 95% CI)		49.17 (4.70, 48.87-4946)	45.33 (4.31, 45.18-45.48)	46.22 (4.69, 46.07-46.36)	<.001
Hip circumference (cm), mean (SD, 95% CI)		101.86 (9.98, 101.24-102.49)	103.95 (11.66, 103.55-104.35)	103.46 (11.33, 103.12-103.81)	<.001
Handgrip strength (kg), mean (SD, 95% CI)		33.94 (9.55, 33.34-34.54)	20.82 (7.17, 20.58-21.07)	23.85 (9.55, 23.56-24.14	<.001
Gait speed (m/sec), mean (SD, 95% CI)		0.93 (0.32, 0.91-0.96)	0.86 (0.27, 0.85-0.87)	0.88 (0.28, 0.87-0.89)	<.001
Five chair-stands time (seconds), mean (SD, 95% CI)		11.80 (3.63, 11.39-12.22)	12.40 (4.18, 12.14-12.65)	12.26 (4.07, 12.04-12.48)	.03
Sarcopenia diagnosis, % (95% CI)		26.66 (23.91-29.55)	23.48 (22.03-24.97)	24.21 (22.93-25.53)	.041

^a^Based on *t* test except nutritional state and sarcopenia diagnosis, which was based on Pearson Chi-square test.

## Discussion

### Principal Findings

In this study, we developed and validated a software for the diagnosis of sarcopenia using an adapted version of the consensus diagnostic criteria [3] and cut-off points for the Chilean population, which can be used with an anthropometric equation or with DXA scan measures [25]. Recently, Brunix et al [32] reviewed several methods to estimate dual muscle mass and concluded that the DXA scan can be considered the reference standard for measuring muscle mass.

Sarcopenia is highly prevalent [9], and its prevalence varies by the definition used. Cruz et al [15] reported a variation from 1% to 29% in elderly community-dwelling populations and from 14% to 33% in long-term care populations using the EWGSOP definition. In Chile, the prevalence of sarcopenia is high (19.1%) and dramatically increases with age, from 12.3% in those 60 to 64 years of age to 38.5% in subjects ≥80 years of age (estimated in a sample of 1006 older adults with DXA scan measures) [25].

Ethgen et al [37] estimated the prevalence of sarcopenia in the next 30 years with a projection model based on the current prevalence of sarcopenia and the demographics available for the populations of 28 countries of the European community, using the lowest and highest estimates. They found that the number of patients with sarcopenia and the prevalence of sarcopenia were projected to increase for the lowest and highest estimates from 2016 to 2045 (11.1%-12.9% and 20.2%-22.3%, respectively), so these results can be relevant in guiding the implementation of public policies.

With respect to the beta and release tests, the viability of the software was demonstrated. The time required to use the software is short, about 11 minutes. In the release test, a large sample was diagnosed in the primary health care centers (4242 community-dwelling people 60 years and older).

As expected in our research, we found that individuals with sarcopenia were in worse physical condition than those without sarcopenia. Patients with sarcopenia have a higher risk of functional limitations, falls, and a decrease in strength and physical performance than robust persons. Several studies have shown the adverse effects of this syndrome on the health and quality of life of older adults [11,12,38-42]. The study conducted by Roth et al [42] showed that the rate of both physical and functional disability is two to three times higher in the population with sarcopenia. Morley, Anker, and von Haehling [16] concluded that sarcopenia was one of the main causes of falls and functional limitations in older people, so it is necessary to screen sarcopenia and treat it.

The low accessibility of the DXA test in primary health care requires the use of low-cost tools and easy management similar to HTSMayor.

In line with the WHO statement of integrated care [43,44] in Chile, the preventive medical examination (EMPAM for its acronym in Spanish) is guaranteed for older people and is ascribed to in the public and private health care systems [45,46]. The EMPAM is a test performed once a year for any adult over 65 years of age, the purpose of which is to investigate (in a timely manner) their functionality and autonomy (eg, the ability of older adults to control their lives, to make their own decisions, and to develop their daily activities). This examination then allows for the identification of risk factors that may endanger the autonomy and independence of an older adult. In this way, anticipatory actions can be planned and carried out by the health care team. Considering the importance of the early diagnosis of sarcopenia, the MINSAL will incorporate the screening of sarcopenia by using HTSMayor at the primary care level. There are few software packages that are used in primary health care, which are mainly used for mental health [47-49].

Our results are a contribution to public health for the older adult population, because it is greatly beneficial to have a valid and safe indicator for the diagnosis of sarcopenia based on anthropometric measurements and strength tests, such as dynamometry and physical performance tests (eg, walking speed for 3 meters or five chair-stands), that are easy to obtain, low in cost, can be replicated in several countries, and can be used for older people in primary health care centers, which represents a growing vulnerable population. For this population, an opportune diagnosis will improve quality of life and avoid the risk factors that are associated with this geriatric syndrome.

### Limitations

A limitation of this study is the low number of incident cases of sarcopenia (37 of 374 people, 9.9% of the patients without sarcopenia at baseline) in the studied period, but the figures are similar to those found by Mijnarends et al [50]. The difference in the frequency of sarcopenia in men and women found in the release version is higher than the one found in the validation study sample, but this situation can be explained considering that the release version was tested in people attending primary care health centers. Another limitation could be the lower sensitivity in women as compared to men. This probably can be explained by the lower accuracy of anthropometric measurements, considering the different fat mass proportion and distribution, specifically hip circumference, in women as compared to men. However, the sensibility in the forms is good enough for the screening of sarcopenia. We do not rule out future upgrades to improve test accuracy.

### Strengths

Among the strengths of our study is the replacement of a DXA scan test by HTSMayor, allowing the diagnosis of sarcopenia in primary health care centers with valid, reliable, low-cost, and easy-to-use software that can be used by the health care team from a mobile device or a computer, which will facilitate the work of clinicians. The availability of this diagnostic tool allowed the development of a Clinical Practice Guide of Sarcopenia for its use at the MINSAL network. This study can be reproduced by other researchers, using prediction equations and cut-off points for their population, which will allow the development of diagnostic instruments for sarcopenia for use in clinical practice.

### Conclusion

We developed and validated a software for the diagnosis of sarcopenia in older Chilean adults that can be used on a mobile device or a computer with good sensitivity and specificity, thus allowing for the development of programs for the prevention, delay, or reversal of this syndrome. The HTSMayor is low in cost and user-friendly. The HTSMayor can be used by health staff to diagnose sarcopenia as part of the preventive medical exam for older adults in public health care centers. To our knowledge, HTSMayor is the first software designed and validated to diagnose sarcopenia.

## Appendix

Multimedia Appendix 1 HTSMayor software demonstration.

Multimedia Appendix 2 Screenshots from app and web.

## Abbreviations

ADL: activities of daily living
ASM: appendicular skeletal muscle mass
DXA: dual-energy x-ray absorptiometry
EMPAM: preventive medical examination
EWGSOP: European Working Group on Sarcopenia in Older People
IADL: instrumental activities of daily living
MINSAL: Ministry of Health of Chile
SMI: skeletal muscle mass index
WHO: World Health Organization.
