# Supplementary material for: Software for the Diagnosis of Sarcopenia in Community-Dwelling Older Adults: Design and Validation Study
Source: JMIR Med Inform. 2020 Apr 13;8(4):e13657. doi: 10.2196/13657 (PMC7186874; doi:10.2196/13657)

Figure 3. Screenshots from HTSmayor app

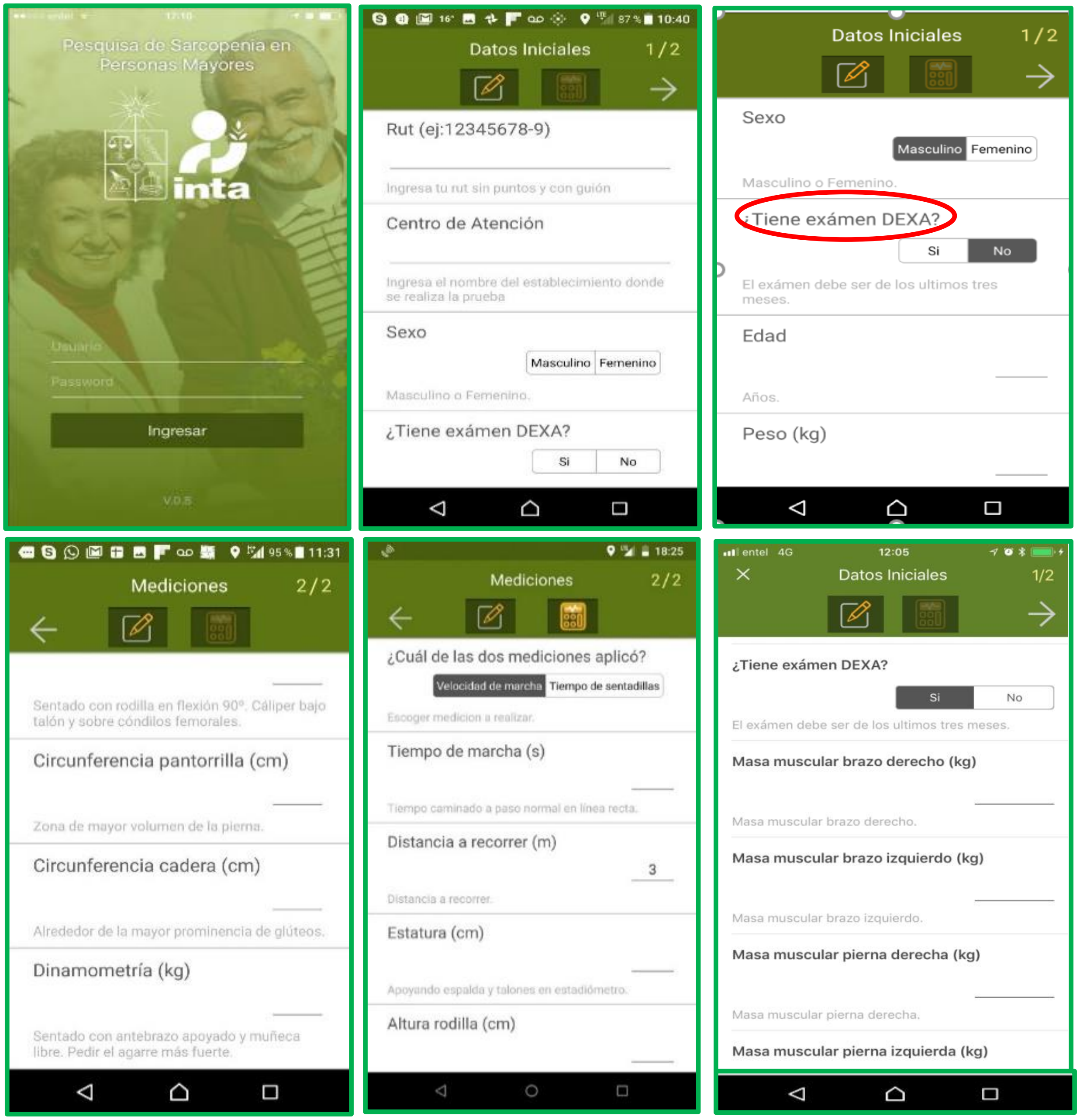

Figure 4. Screenshots from HTSmayor (Web version)

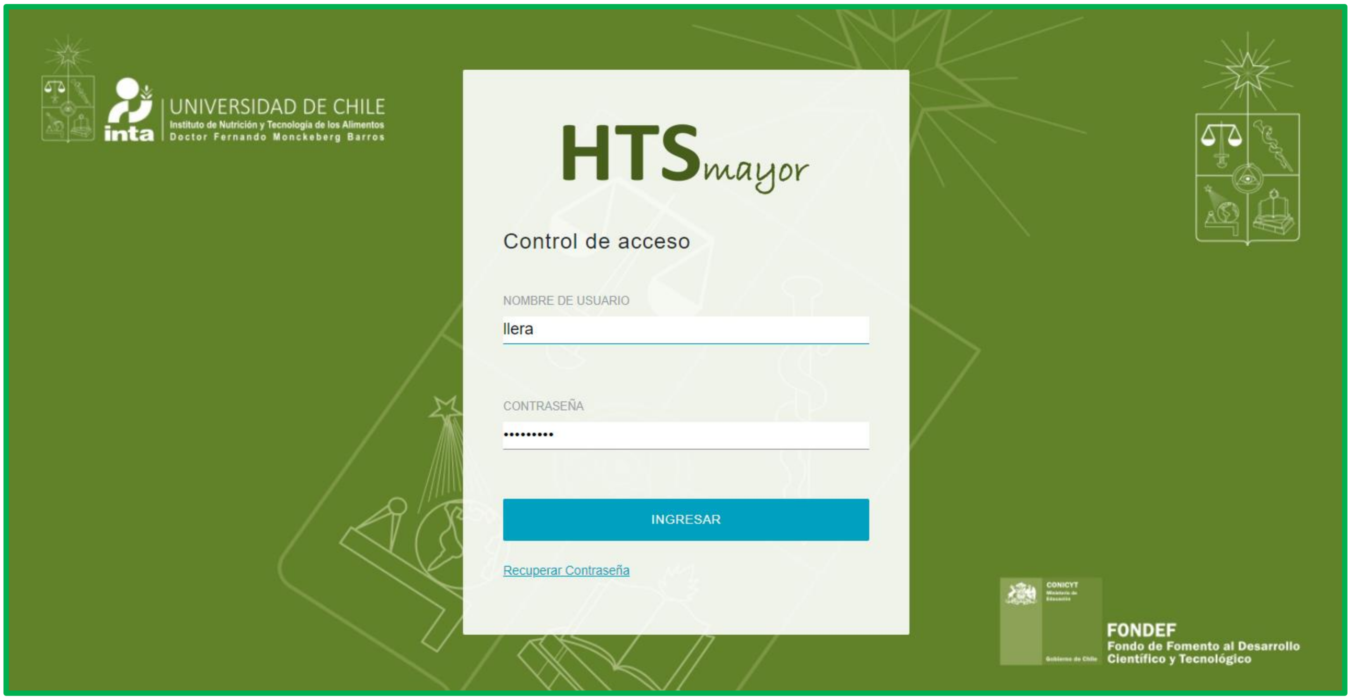

Figure 5. Results of the HTSmayor (mobile app and Web versions)

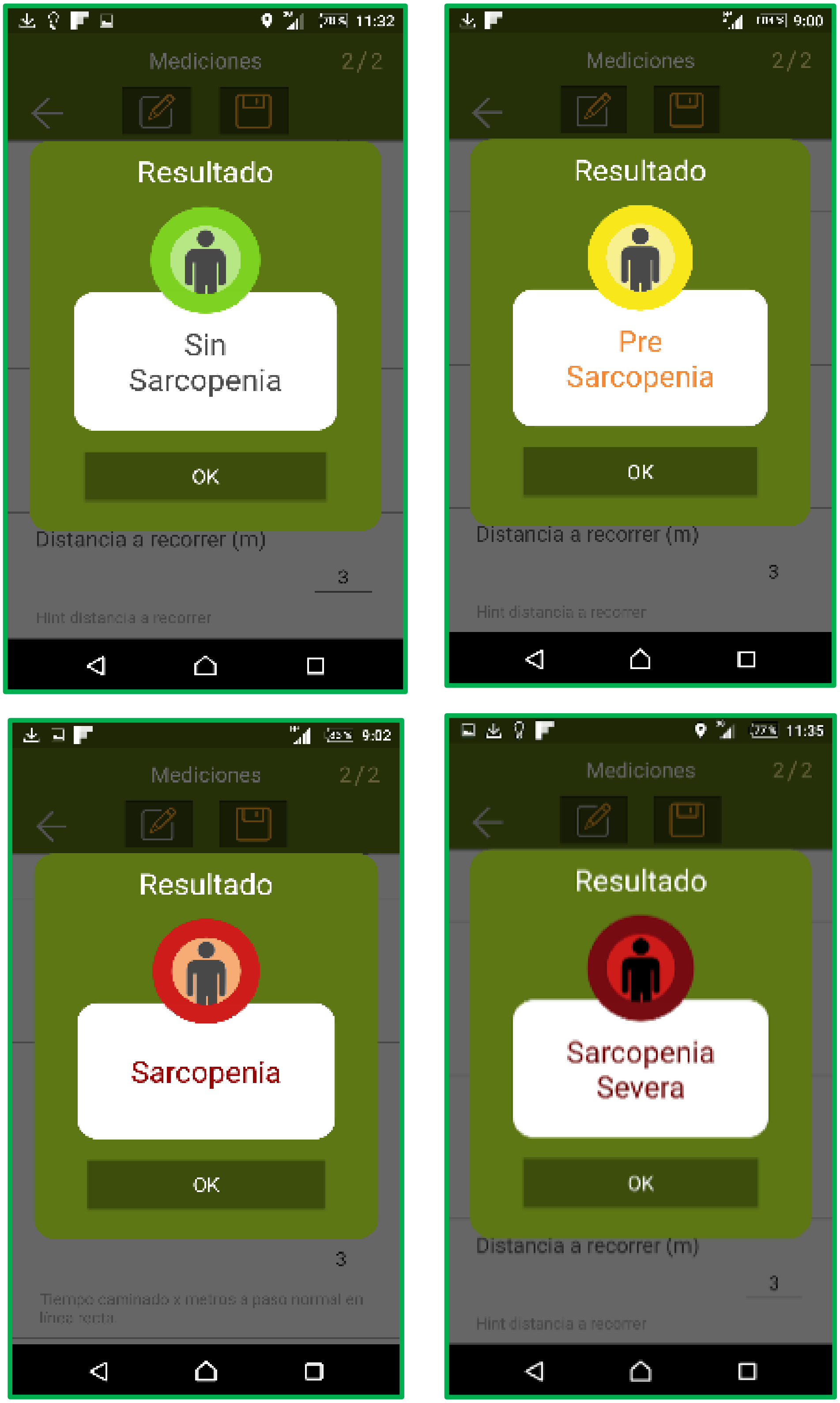

Supplement: Multimedia Appendix 2 [file medinform_v8i4e13657_app2.pdf]
